# Quality of life improvements following one year of setmelanotide in children and adult patients with Bardet–Biedl syndrome: phase 3 trial results

**DOI:** 10.1186/s13023-022-02602-4

**Published:** 2023-01-16

**Authors:** Elizabeth Forsythe, Robert M. Haws, Jesús Argente, Philip Beales, Gabriel Á. Martos-Moreno, Hélène Dollfus, Costel Chirila, Ari Gnanasakthy, Brieana C. Buckley, Usha G. Mallya, Karine Clément, Andrea M. Haqq

**Affiliations:** 1 Genetics and Genomics Medicine Programme, University College London Great Ormond Street Institute of Child Health, London, UK; 2 Marshfield Clinic Research Institute, Marshfield, WI USA; 3 Department of Pediatrics and Pediatric Endocrinology, Universidad Autónoma de Madrid, University Hospital Niño Jesús, Madrid, Spain; 4 CIBER “Fisiopatología de la Obesidad y Nutrición” (CIBEROBN), Instituto de Salud Carlos III, Madrid, Spain; 5 IMDEA Food Institute, Madrid, Spain; 6 Hôpitaux Universitaires de Strasbourg, CARGO and Department of Medical Genetics, Strasbourg, France; 7 RTI Health Solutions, Research Triangle Park, NC USA; 8 Rhythm Pharmaceuticals, Inc., Boston, MA USA; 9 Assistance Publique-Hôpitaux de Paris, Nutrition Department, Pitié-Salpêtrière Hospital, Paris, France; 10 Sorbonne Université, INSERM, NutriOmics Research Unit, Paris, France; 11 Division of Pediatric Endocrinology, University of Alberta, 6-002E Li Ka Shing Centre for Health Research Innovation, Edmonton, AB T6G 2E1 Canada

**Keywords:** BBS, Genetic obesity, IWQOL-Lite, PedsQL, Quality of life, Setmelanotide

## Abstract

**Background:**

Bardet–Biedl syndrome is a rare genetic disease associated with hyperphagia and early-onset, severe obesity. There is limited evidence on how hyperphagia and obesity affect health-related quality of life in patients with Bardet–Biedl syndrome, and on how management of these symptoms may influence disease burden. This analysis evaluated changes in health-related quality of life in adults and children with Bardet–Biedl syndrome in a Phase 3 trial following 1 year of setmelanotide treatment (ClinicalTrials.gov identifier: NCT03746522).

**Methods:**

Patients with Bardet–Biedl syndrome and obesity received 52 weeks of treatment with setmelanotide and completed various self-reported health-related quality of life measures. Patients aged < 18 years or their caregiver completed the Pediatric Quality of Life Inventory (PedsQL; meaningful improvement, 4.4-point change); adults aged ≥ 18 years completed the Impact of Weight on Quality of Life Questionnaire-Lite (IWQOL-Lite; meaningful improvement range, 7.7–12-point change). Descriptive outcomes were reported in patients with data both at active treatment baseline and after 52 weeks of treatment.

**Results:**

Twenty patients (< 18 years, n = 9; ≥ 18 years, n = 11) reported health-related quality of life at baseline and 52 weeks. For children and adolescents, PedsQL score mean change from baseline after 52 weeks was + 11.2; all patients with PedsQL impairment at baseline (n = 4) experienced clinically meaningful improvement. In adults, IWQOL-Lite score mean change from baseline was + 12.0. Of adults with IWQOL-Lite impairment at baseline (n = 8), 62.5% experienced clinically meaningful improvement. In adults, IWQOL-Lite score was significantly correlated with changes in percent body weight (*P* = 0.0037) and body mass index (*P* = 0.0098).

**Conclusions:**

After 1 year of setmelanotide, patients reported clinically meaningful improvements across multiple health-related quality of life measures. This study highlights the need to address the impaired health-related quality of life in Bardet–Biedl syndrome, and supports utility of setmelanotide for reducing this burden.

*Trial Registration* NCT03746522. Registered November 19, 2018, https://clinicaltrials.gov/ct2/show/NCT03746522.

## Background

Bardet–Biedl syndrome (BBS) is a rare genetic disease of obesity characterized by dysfunction of the primary cilia and associated with multiorgan dysfunction [1, 2]. The prevalence of BBS varies across populations, with estimates ranging from 1:100,000 to 1:160,000 across Europe and North America [2, 3]. BBS is inherited in an autosomal recessive manner and is associated with variants in ≥ 25 genes [4–6]. A clinical diagnosis is made on the basis of the presence of primary and secondary features, and is confirmed molecularly in ~ 80% of cases [1, 7]. The primary diagnostic features of BBS are retinal degeneration (93%), obesity (72–92%), postaxial polydactyly (63–81%), genital anomalies (59–98%), renal anomalies (53%), and learning difficulties (61%) [7]. Hyperphagia—an insatiable and pathological hunger—and obesity are hallmark symptoms of BBS [7–9]. Most patients with BBS have symptoms of hyperphagia in the first years of life [8]. Approximately 70% of patients experience obesity or overweight by the age of 5 years, and ≥ 90% over the age of 6 years [10].

The hyperphagia and resulting obesity manifesting in individuals with BBS are associated with impaired signaling in the central melanocortin pathway of the hypothalamus, which is a critical component in the control of energy intake and expenditure [11, 12]. BBS genes are required for leptin receptor (LEPR) trafficking, a key component of the melanocortin pathway. Under normal conditions, leptin binds the leptin receptor on proopiomelanocortin (POMC) neurons, and POMC is then cleaved by the protein encoded by *PCSK1*. POMC cleavage generates α–melanocyte-stimulating hormone, which activates melanocortin-4 receptor (MC4R) leading to decreased food consumption [11–13]. Mouse models lacking specific key BBS proteins develop hyperphagia and obesity associated with reduced LEPR signaling and mistrafficking of the receptor to the plasma membrane [14, 15].

Historically, treatment strategies for BBS were primarily symptomatic [2, 16, 17]. Recommendations for management of BBS and other syndromic forms of obesity highlight the importance of multidisciplinary care and genetic counseling [2, 18, 19]. Genetic diagnosis of BBS and subsequent genetic counseling can also inform management strategies [18, 19]. Prior to the availability of targeted treatment, traditional recommendations for weight management in patients with BBS and other syndromic forms of obesity were similar to those for the general population and included lifestyle modifications and eventual bariatric surgery, although data on long-term outcomes are limited [2, 16, 18, 20, 21].

Extensive research in the general population has demonstrated that obesity poses a substantial physical and psychosocial burden for patients and caregivers [22–28]. Individuals with obesity are at a much greater risk of morbidity (e.g., diabetes, cardiovascular disease, chronic kidney disease, respiratory dysfunction, reduced functional mobility) and mortality [29]. While there is a wide breadth of evidence supporting the impact of general obesity on quality of life, there is limited evidence for the effects of hyperphagia and obesity on quality of life in patients with BBS. One study in caregivers of patients with BBS found that obesity stigmatization is common in this population, similar to stigmatization experienced by patients with general obesity [22, 30]. In patients with other rare genetic diseases of obesity, such as POMC, proprotein convertase subtilisin/kexin type 1 (PCSK1), or LEPR deficiency, notable impairments in quality of life have been reported [31]. Hyperphagia can also contribute to impaired quality of life. Individuals with self-reported hyperphagia and severe obesity report much lower quality of life than the general population [32]. In patients with obesity due to BBS, POMC deficiency, or LEPR deficiency, hyperphagia is associated with impaired health-related quality of life (HRQOL), with patients reporting guilt, frustration, sadness, and feelings of failure given the inability to control their hunger [33, 34]. These patients and their caregivers also reported a negative impact on family dynamics and performance at work or in school, attributed to the symptoms of hyperphagia [33, 34]. Given the early onset of hyperphagia and obesity, most patients with BBS and their families have likely lived with the burdens associated with these symptoms for most of their lives [8, 10, 30].

Because of the limited treatment options for hyperphagia and obesity, there is a need for evidence regarding the impact of weight loss interventions on quality of life in patients with BBS. In Phase 2 and 3 trials of patients with BBS, the MC4R agonist setmelanotide has demonstrated clinically significant reductions in body weight and hunger scores [35, 36]. In the Phase 3 trial, meaningful reductions in weight and body mass index (BMI) Z score were observed after 1 year of setmelanotide treatment (− 7.6% weight change from baseline in those ≥ 18 years old; − 0.75-point BMI Z score change from baseline in those < 18 years old). Treatment was associated with clinically beneficial reductions in mean BMI in patients ≥ 18 years old (− 9.1%) and < 18 years old (− 9.5%) [36]. Importantly, setmelanotide treatment also led to significant reductions in hunger scores in patients able to self-report hunger (− 30.5% change from baseline; *P* = 0.0004). These results led to the approval of setmelanotide by the US Food and Drug Administration in 2022 for chronic weight management in BBS [37].

There is an unmet need for evidence on quality of life in patients with BBS and obesity and on how hyperphagia and obesity management affect HRQOL. To address this, the objective of this sub-study of the Phase 3 trial of setmelanotide was to evaluate changes in HRQOL in adults and children with BBS following 1 year of treatment with setmelanotide.

## Methods

### Study design

A multicenter Phase 3 trial that included a randomized, double-blind, placebo-controlled period evaluated 52 weeks of treatment with setmelanotide in patients with BBS and obesity (ClinicalTrials.gov identifier: NCT03746522) [17]. Full details of the trial design have been published previously [17]. Eligible patients were ≥ 6 years of age with a clinical diagnosis of BBS or Alström syndrome and obesity, defined as BMI ≥ 30 kg/m^2^ (patients aged ≥ 16 years) or weight > 97th percentile (patients aged 6 to < 16 years). While a cohort of patients with Alström syndrome was enrolled, the current analysis focuses on the population of patients with BBS. This trial was conducted according to standards set by the International Council on Harmonisation for Good Clinical Practice, the Declaration of Helsinki, and all applicable regulatory requirements. All participating study sites had obtained institutional review board approval. Patients or guardians provided written informed consent.

Patients were randomized to receive either setmelanotide or placebo during a 14-week, double-blind period with a dose escalation to 3.0 mg of setmelanotide (or placebo equivalent), followed by open-label setmelanotide for a total of ≥ 52 weeks setmelanotide treatment [17]. Efficacy was assessed in all patients who receive ≥ 1 dose of setmelanotide and had baseline data; safety was assessed in all patients receiving ≥ 1 dose of setmelanotide or placebo.

### Endpoints and assessments

The primary endpoint was the proportion of patients aged ≥ 12 years who reached ≥ 10% reduction in body weight compared with baseline after 52 weeks of setmelanotide treatment [17]. Key secondary endpoints included percent change in body weight and hunger scores after 52 weeks of setmelanotide, and the proportion of patients ≥ 12 years with ≥ 25% improvement in hunger scores after 52 weeks. A psychometric evaluation estimating the threshold of meaningful within-patient change was determined to be ≥ 1- and ≥ 2-point change (unpublished data; Rhythm Pharmaceuticals). Body weight, height, vital signs, and concomitant medications were reviewed during on-site visits throughout the trial. Hunger scores were evaluated in patients ≥ 12 years old without cognitive impairment. Patients completed a daily hunger questionnaire using a Likert-type scale ranking morning hunger, most hunger, and average hunger over the past day.

Self-reported HRQOL was measured in pediatric and adolescent patients (aged ≥ 6 to < 18 years) by patients or caregivers using age-specific Pediatric Quality of Life Inventory (PedsQL) assessments (child age range, 5–12 years; teen age range, 13–18 years) [38]. The PedsQL is a 23-item, self- or caregiver-reported, age-dependent assessment of HRQOL in children and adolescents with or without acute or chronic health conditions that encompasses 4 domain scores (physical, emotional, social, and school functioning) [39, 40]. In this analysis, self-reported data for PedsQL were utilized. For adults (≥ 18 years), HRQOL was self-reported using the validated, obesity-specific Impact of Weight on Quality of Life Questionnaire-Lite (IWQOL-Lite). The IWQOL-Lite questionnaire is a 31-item, obesity-specific assessment of HRQOL consisting of a total score and 5 domain scores (physical function, self-esteem, public distress, sex life, work) [41]. Raw scores for both IWQOL-Lite and PedsQL are transformed on a scale of 0–100, with 0 representing the worst possible and 100 the best possible HRQOL [40, 41]. Comparative populations without obesity have a mean (SD) PedsQL total score of 83.0 (14.8) and a mean (SD) IWQOL-Lite total score of 94.7 (7.6) [27, 41]. HRQOL impairment is defined on the basis of PedsQL or IWQOL-Lite total scores. PedsQL impairment is defined as < 68.2 for PedsQL total score [27]. For IWQOL-Lite, impairment thresholds vary from mild (range, 79.5–87.0), to moderate (range, 71.9–79.4), to severe (< 71.8) [41]. Clinically meaningful improvement thresholds are defined as 4.4 for PedsQL total score [40] and range from 7.7 to 12 (depending on baseline score) for IWQOL-Lite total score [41]. Patients aged 5–12 years used the PedsQL-Child assessment; patients aged 13–18 years used the PedsQL-Teen assessment. Patients ≥ 18 years were given the IWQOL-Lite assessment.

This analysis focuses on the cohort of patients with BBS. Descriptive analyses were conducted on outcomes reported for patients with either PedsQL or IWQOL-Lite data both at active treatment baseline, defined as the last available measure before the first active setmelanotide dose, and after 52 weeks of setmelanotide treatment. Spearman correlations were used to evaluate associations between percent change in PedsQL or IWQOL-Lite and changes in body weight, BMI or BMI Z score, and hunger scores.

## Results

### Patient demographics, disposition, and weight outcomes at week 52

**Fig. 1:**
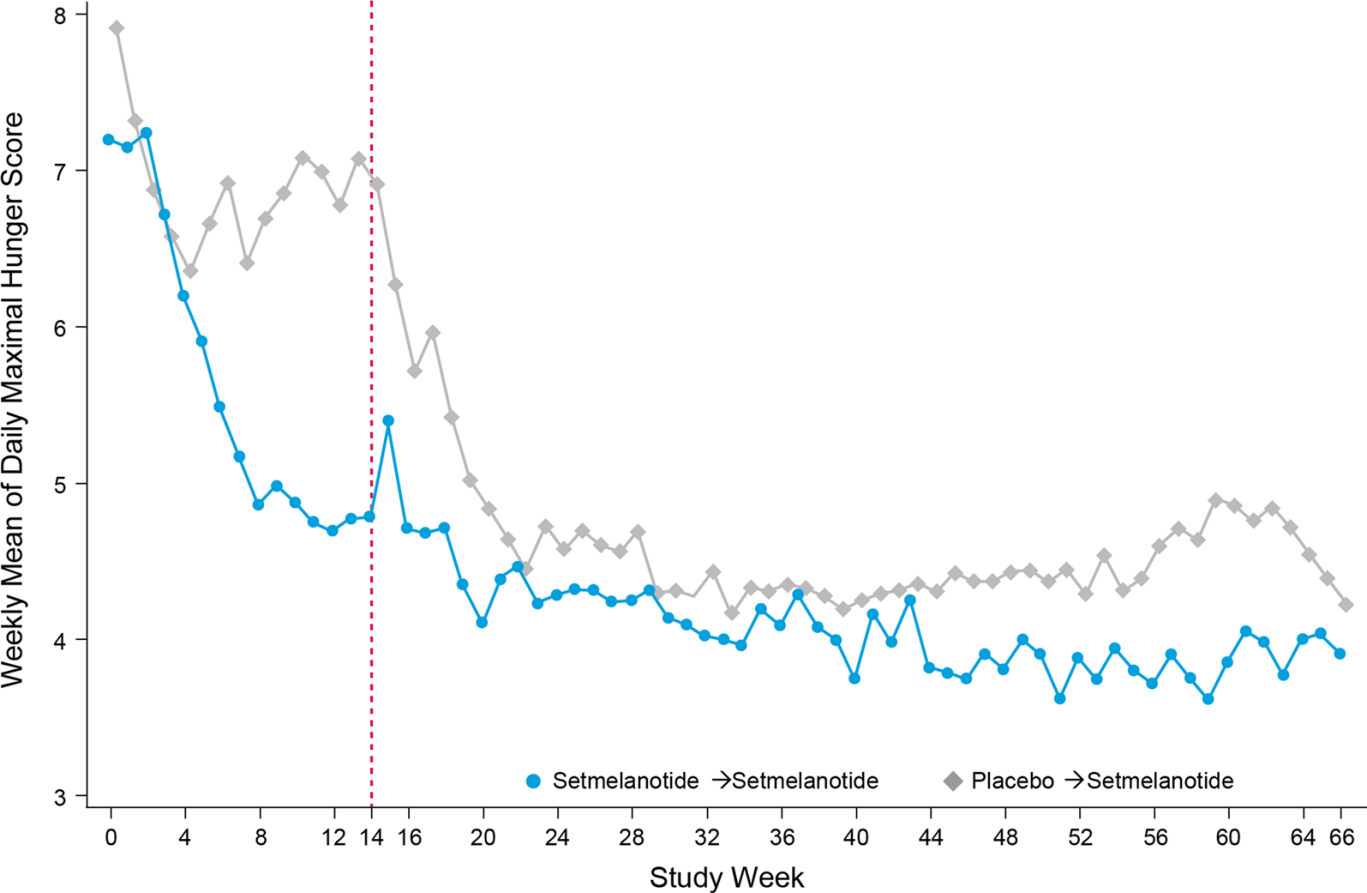
Change in maximal hunger score over time. Weekly mean of the daily maximal hunger score over time across all patients enrolled in the Phase 3 trial with BBS who could self-report hunger (i.e., ≥ 12 years old without cognitive impairment). Patients were randomized to receive 14 weeks of setmelanotide (n = 5 at baseline) or placebo (n = 9 at baseline), followed by 52 weeks of open-label setmelanotide (n = 14 after 52 weeks). Vertical dashed pink line represents the Week-14 time point where all patients transitioned to open-label setmelanotide treatment

Patients were enrolled between December 10, 2018, and November 25, 2019. Of 32 patients with BBS enrolled in the study, 31 received ≥ 1 dose of study drug [36]. Across all patients ≥ 12 years without cognitive impairment reporting hunger, reductions in the weekly mean of daily hunger score were observed in patients receiving setmelanotide (n = 5) compared with placebo (n = 9) in a 14-week, double-blind, placebo-controlled phase; reductions in hunger were sustained over the full study course, with patients originally receiving placebo showing rapid reduction in hunger scores following setmelanotide initiation (Fig. 1). Of these, 20 patients (65% female, 85% White), including 10 without cognitive impairment, had HRQOL data at baseline and Week 52 and were therefore included in this analysis (< 18 years old, n = 9; ≥ 18 years old, n = 11; Table 1). All patients had genetic confirmation validating a clinical diagnosis of BBS. The mean (range) age and BMI of the 20 evaluable patients at baseline were 22 (10–44) years and 43.4 (24.4–61.4) kg/m^2^, respectively. Mean (range) BMI for patients < 18 and ≥ 18 years was 38.3 (24.4–61.3) kg/m^2^ and 47.6 (39.5–57.8) kg/m^2^, respectively. The mean age at diagnosis was 9 years. Baseline data were comparable to the overall enrolled study population (N = 32; mean age, 20.2 years; mean BMI, 41.6 kg/m^2^). [36]

**Table 1:** Baseline characteristics

	All patients^a^	Patients without cognitive impairment
Patients who reported HRQOL assessments, n	20	10
Age, mean (SD) [range], years	22.0 (10.9) [10–44]	23.3 (10.7) [12–43]
Female sex, % [n]	65 (13)	70 (7)
BMI, mean (SD) [range], kg/m^2^	43.4 (9.6) [24.4–61.4]	45.3 (10.7) [24.4–57.8]
≥ 18 years	47.6 (5.8) [39.4–57.8]	50.2 (5.3) [43.0–57.8]
< 18 years	38.3 (10.7) [24.4–61.4]	34.8 (8.0) [24.4–49.1]
Maximal hunger score, mean (SD)^b^	–	6.8 (1.2)

^a^Includes adults and children with and without cognitive impairment. Includes randomized patients who received ≥ 1 dose of setmelanotide or placebo and have baseline and Week 52 data. ^b^Hunger was evaluated in patients ≥ 12 years without cognitive impairment

*BMI* body mass index, *HRQOL* health-related quality of life, *SD* standard deviation

After 52 weeks of treatment, mean change in BMI Z score in patients < 18 years old (n = 9) was − 0.7 points (Table 2). In patients ≥ 18 years old (n = 11), mean BMI after 52 weeks changed − 9.4% from baseline (Table 3).

**Table 2:** Impact of setmelanotide in children (< 18 years old; self-reported) with baseline and week-52 PedsQL data

	Baseline		Change from baseline at week 52	
	All patients (n = 9)	Patients without cognitive impairment (n = 3)	All patients (n = 9)	Patients without cognitive impairment (n = 3)
PedsQL total score, mean (SD) [range]	67.2 (18.9) [33.7–90.2]	83.3 (2.7) [80.4–87.0]	+ 11.2 (14.3) [− 5.2 to 45.6]	+ 3.3 (6.6) [− 5.2 to 10.9]
PedsQL physical function score, mean (SD) [range]	60.4 (28.1) [0–93.8]	83.3 (7.8) [75.0–93.8]	+ 14.0 (27.7) [− 18.8–68.8]	+ 2.1 (14.7) [− 18.8–12.5]
PedsQL psychosocial score, mean (SD) [range]	70.7 (16.3) [46.7–90.0]	83.3 (5.4) [76.7–90.0]	+ 9.3 (9.9) [0.0–33.3]	+ 3.9 (4.4) [0.0–10.0]
BMI Z-score, mean (SD) [range]	3.7 (1.5) [1.8–7.1]	2.9 (1.4) [1.8–4.8]	− 0.7 (0.5)^a^ [− 0.2 to − 1.9]	− 1.0 (0.7)^a^ [− 0.2 to − 1.9]
Maximal hunger, mean (SD) [range]	–	6.0 (0.8) [5.0–7.0]	–	− 52.7% (23.2) [− 71.4% to − 20.0%]

^a^Absolute change. *BMI* body mass index, *PedsQL* pediatric quality of life inventory, *SD* standard deviation

**Table 3:** Impact of setmelanotide in adults (≥ 18 years old; self-reported) with baseline and week-52 IWQOL-Lite data

	Baseline		Change from baseline at week 52	
	All patients (n = 11)	Patients without cognitive impairment (n = 7)	All patients (n = 11)	Patients without cognitive impairment (n = 7)
IWQL-Lite total score, mean (SD) [range]	74.9 (12.0) [59.0–97.0]	70.7 (9.8) [59.0–88.0]	+ 12.0 (10.3) [1.0–28.0]	+ 17.6 (8.9) [1.0–28.0]
IWQOL-Lite physical function score, mean (SD) [range]	63.0 (13.3) [48.0–95.0]	60.0 (7.8) [52.0–73.0]	+ 15.3 (11.6) [0.0–34.0]	+ 21.7 (9.3) [5.0–34.0]
IWQOL-Lite sexual life score, mean (SD) [range]	90.1 (14.2) [63.0–100.0]	86.7 (15.7) [63.0–100.0]	+ 9.3 (13.4) [0.0–37.0]	+ 12.4 (14.8) [0.0–37.0]
IWQOL-Lite work score, mean (SD) [range]	83.7 (16.2) [50.0–100.0]	77.9 (17.6) [50.0–100.0]	+ 9.5 (14.0) [− 6.0 to 37.0]	+ 15.0 (14.8) [0.0–37.0]
IWQOL-Lite public distress score, mean (SD) [range]	75.0 (17.7) [50.0–100.0]	68.6 (13.6) [50.0–95.0]	+ 12.7 (15.0) [− 5.0 to 40.0]	+ 20.0 (14.1) [− 5.0 to 40.0]
IWQOL-Lite self-esteem score, mean (SD) [range]	79.1 (19.1) [32.0–100.0]	74.3 (21.3) [32.0–100.0]	+ 11.1 (15.9) [0.0–50.0]	+ 15.4 (18.4) [0.0–50.0]
BMI, kg/m^2^, mean (SD) [range]	47.6 (5.8) [39.4–57.8]	50.3 (5.3) [43.0–57.8]	− 9.4% (6.7) [− 17.6 to 5.3%]	− 10.1% (7.4) [− 17.6 to 5.3%]
Maximal hunger, mean (SD) [range]	–	7.1 (1.2) [5.0–9.0]	–	− 39.6% (21.5) [− 71.4 to 0%]

*BMI* body mass index, *IWQOL-Lite* impact of weight on quality of life-lite, *SD* standard deviation

### Children and adolescent patients

For children and adolescents (< 18 years old; n = 9) with BBS, mean (range) PedsQL total score at active treatment baseline was 67.2 (33.7–90.2; Table 2). Across all patients, an improvement in mean PedsQL total score was observed, regardless of baseline impairment (n = 9). Mean (range) change in PedsQL total score was + 11.2 (− 5.2 to 45.6) across all evaluable patients, and + 3.3 (− 5.2 to 10.9) in those without cognitive impairment. Improvements in PedsQL physical function and psychosocial scores were also seen after 52 weeks; mean (range) improvements in physical function and psychosocial scores were + 14.0 (− 18.8 to 68.8) and + 9.3 (0–33.3), respectively.

**Fig. 2:**
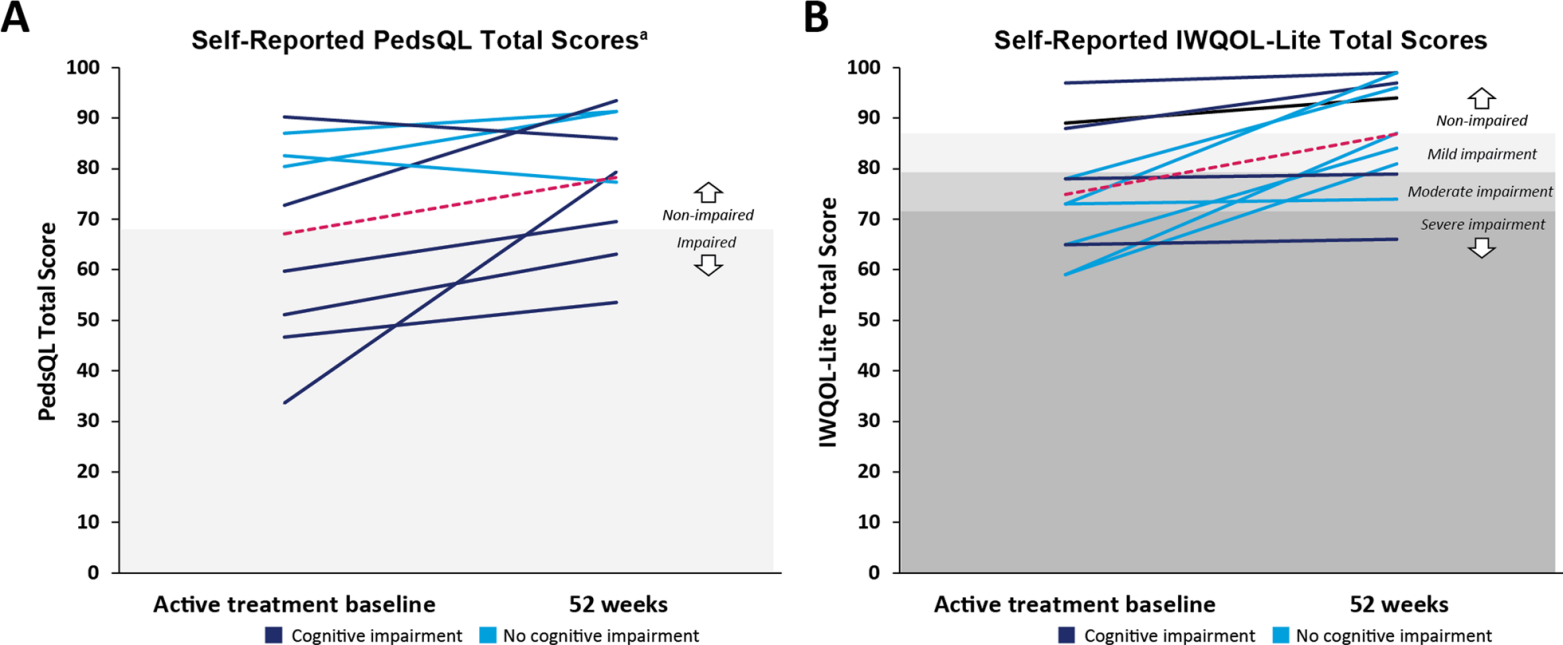
Change in PedsQL and IWQOL-Lite scores after 52 weeks. Spaghetti plots of individual patient HRQOL course in **A** pediatric and adolescent patients (n = 9) and **B** adults (n = 11) from active treatment baseline to 52 weeks of treatment. Solid blue lines represent patient-level scores. Dashed pink lines represent the group mean at each visit. Shading indicates threshold for HRQOL impairment (PedsQL: impairment < 68.2; IWQOL-Lite: mild impairment range 79.5–87.0, moderate impairment range 71.9–79.4, severe impairment < 71.8). Only patients with active treatment baseline and 52-week data were included. ^a^Includes patients who reported PedsQL-Child or PedsQL-Teen. HRQOL, health-related quality of life; IWQOL-Lite, Impact of Weight on Quality of Life Questionnaire-Lite; PedsQL, Pediatric Quality of Life Inventory

Overall, 4 of 9 (44.4%) children experienced impairment in HRQOL at active treatment baseline (mean [SD] total score, 47.8 [10.9], n = 4). All children with HRQOL impairment based on PedsQL at active treatment baseline (n = 4) experienced clinically meaningful improvement after 52 weeks of treatment (Fig. 2A). Mean (range) improvement in PedsQL total score was + 18.6 (6.8–45.6) in those with HRQOL impairment at baseline. Improvements in mean (range) physical function (+ 21.4 [− 17.7 to 68.8]) and psychosocial (+ 16.2 [5.0–33.3]) scores were also observed at Week 52 in patients with PedsQL HRQOL impairment at baseline. All children without clinically relevant PedsQL HRQOL impairment at active treatment baseline either preserved their non-impaired status (n = 3; 1 with cognitive impairment, 2 without) or further enhanced their status and reported meaningful improvements in their PedsQL total score (n = 2; 1 with cognitive impairment, 2 without) (Fig. 2A). Mean (range) change in PedsQL total score in those without PedsQL HRQOL baseline impairment was + 5.2 (− 5.2 to 20.6). Among patients without baseline PedsQL impairment (n = 5), mean (range) changes in physical function and psychosocial scores were + 8.1 (− 18.8 to 46.9) and + 3.7 (0–10), respectively.

### Adult patients

Among adult patients (≥ 18 years old; n = 11), the mean (range) IWQOL-Lite total score at active treatment baseline was 74.9 (59.0–97.0), indicating moderate HRQOL impairment (Table 3). Mean (range) improvement in IWQOL-Lite total score was + 12.0 (1.0–28.0) in all evaluable patients (n = 11), and + 17.6 (1.0–28.0) in those without cognitive impairment (n = 7; Table 3, Fig. 2B).

At the patient level, 8 of 11 (72.7%) adults experienced HRQOL impairment at active treatment baseline (mean [range], 68.8 [59.0–78.0]). Of these, 4 adults reported severe HRQOL impairment, and 4 reported moderate HRQOL impairment. Of adults with HRQOL impairment at active treatment baseline, 5 of 8 (62.5%) experienced clinically meaningful improvement in IWQOL-Lite and improved their health status level of impairment after 52 weeks of treatment; notably, 2 patients reporting moderate impairment at baseline improved to non-impaired status at the end of the study. The 3 remaining patients preserved their HRQOL, demonstrating improvements in total IWQOL-Lite score that did not meet the threshold for being clinically meaningful. Mean (range) change in total IWQOL-Lite scores in those with impairment at baseline (n = 8) was + 14.5 (1.0–28.0). All adults without clinically relevant HRQOL impairment at active treatment baseline (n = 3) either preserved their non-impaired status (n = 2) or meaningfully improved (n = 1) their HRQOL based on IWQOL-Lite scores. Mean (range) change in total IWQOL-Lite scores in those without impairment at baseline (n = 3) was + 5.3 (2.0–9.0).

### Correlations between HRQOL and efficacy outcomes

In adult patients (n = 11), statistically significant correlations were observed between percent change in IWQOL-Lite and percent change in body weight (Spearman correlation coefficient, − 0.79; *P* = 0.0037) and BMI (− 0.74; *P* = 0.0098). No significant correlations were observed between change in body weight or BMI Z score and PedsQL score, or between either HRQOL assessment and hunger scores in those who self-assessed hunger (i.e., pediatric patients ≥ 12 years old or adults without cognitive impairment). Correlations in the pediatric population were difficult to assess given the limited sample size.

## Discussion

Obesity itself can impose a substantial physical and psychosocial burden for patients and their families [22–28]. The presence of hyperphagia can compound this burden in patients and families living with rare genetic diseases of obesity, including BBS [8, 30, 33]. Setmelanotide targets the impaired MC4R signaling pathway, which controls energy intake, in BBS and therefore may alleviate hyperphagia which, in turn, could lead to impacts on weight and HRQOL [11–13]. In light of the lack of evidence on quality of life in individuals with BBS and obesity, the baseline HRQOL data reported in this study address a literature gap and provide key evidence describing the range of HRQOL impairment in patients with BBS. In this population, 60% of patients reported clinical impairment in HRQOL at baseline. The HRQOL deficits reported at baseline in patients with BBS were similar to those reported by patients with POMC, PCSK1, or LEPR deficiency [31]. Compared with HRQOL reported in clinical trials of the glucagon-like peptide 1 receptor agonist semaglutide in patients with general obesity, patients with BBS reported comparable or worse baseline HRQOL than those with general obesity [42]. Baseline PedsQL scores in children and adolescents were also notably worse than averages reported in patients with other chronic conditions, including general obesity, diabetes, gastrointestinal conditions, cardiac disease, asthma, and cancer [43]. Self-reported outcomes in patients with cognitive impairment can be challenging to interpret. For this reason, HRQOL results are shown for both the overall population and for patients without cognitive impairment. The symptoms of BBS are progressive, evolve over time, and are variable across individuals, creating a challenge for addressing quality of life burdens [7]. In progressive diseases, the burden and effect on overall HRQOL can increase concurrently with disease progression [44, 45]. While patients in this study reported HRQOL impairment at baseline, the level of impairment may have been underestimated because of the genetic nature of the disease, causing patients to have a different threshold for baseline HRQOL compared with the general population. Further, there is a lack of evidence on the natural history of patients with BBS. Therefore, the true HRQOL burden of these patients has not been assessed, and the associated changes in HRQOL, particularly in those who experience hyperphagia early in life and may learn to adapt, may not have been fully captured. Therefore, there is a critical need for targeted intervention to stabilize or improve HRQOL in patients with BBS.

Variability was also observed in baseline HRQOL across patients, as well as in which patients experienced impairment based on different assessments. It is known that response shifting can occur in patients living with chronic disease, where patient perceptions of health shift to cope with their situation [46]. Quality of life measures can therefore be challenging in chronic conditions, as health state is subjective across individuals and may differ based on the assessment used. Further, a single individual may interpret or report a similar score differently throughout their disease course. Additional outside factors may have led to variability in baseline scores, including home and school or work environments, and access to supportive care. A substantial portion of the study for most patients occurred during the global COVID-19 pandemic, which may have acted as a unique modifying factor to HRQOL. A separate contributing factor may have been the proportion of patients with cognitive impairment (50% of patients), which may have influenced self-reported assessments. Nonetheless, the pretreatment baseline scores of patients with BBS highlight the high level of HRQOL burden experienced by patients living with this disease.

This is the first Phase 3 trial to evaluate the impact of setmelanotide treatment on HRQOL in patients with BBS. Most patients with impaired HRQOL at active treatment baseline reported clinically meaningful improvements in HRQOL after 52 weeks of setmelanotide treatment. Improvements were seen in total PedsQL and IWQOL-Lite scores, along with domain scores including psychosocial and physical functioning. Among patients without impaired HRQOL at baseline, most either preserved or improved their status. It is challenging to determine a direct connection between weight loss and HRQOL, particularly in pediatric patients where HRQOL is assessed by PedsQL, which is not specific to obesity-related measurements of HRQOL. A potential link is supported by these data and a separate sub-study, which conducted qualitative interviews following this trial, demonstrating dramatic qualitative improvements in hyperphagia and quality of life reported by patients with BBS and caregivers [34]. Before setmelanotide treatment, patients with BBS experienced poor emotional well-belling, difficulties with concentration that affected school performance, and impaired familial relationships. In addition, 1 patient reported persistent hunger, anticipation of their next meal, and the feeling of unhappiness when they were unable to eat. Both patients and caregivers describe all-consuming hunger associated with BBS that was notably improved with setmelanotide treatment. Following setmelanotide treatment, a patient stated that, on occasion, they would stop eating even if more food was available and they felt satisfied after meals. Patients reported substantial improvements in physical and emotional health with treatment and high levels of treatment satisfaction. For example, another patient reported feeling more positive and described their desire to be more social. Meaningful within-patient changes in hunger score were observed in this cohort and further support the quantitative hunger score data in this study, despite hunger scoring being subjective. Similar impacts of setmelanotide treatment on HRQOL and hyperphagia burden have also been reported in pediatric and adult patients with other rare genetic diseases of obesity, including POMC and LEPR deficiency, highlighting the clinical utility of setmelanotide for HRQOL in patients with rare genetic diseases of obesity. [31, 33]

While the current study did not show significant correlations between quantitative measures of hunger and PedsQL, these qualitative examples of patient experience further support the utility of setmelanotide treatment. Further, ≥ 40% improvement in hunger scores were observed in adult and pediatric patients without cognitive impairment in this study. Results in patients with BBS without cognitive impairment in this study highlight that the benefits of obesity management strategies may extend beyond weight and encompass hunger and HRQOL. A more thorough understanding of the link between HRQOL and weight loss or hunger is needed in this population.

A limitation of this study is the small sample sizes across some of the assessments, which may in part be due to the rarity of the disease. Although improvements in IWQOL-Lite were correlated with BMI reductions in adults, the small sample sizes should be considered when interpreting these results. Similarly, while no significant correlations were observed between weight or hunger outcomes and HRQOL in pediatric and adolescent patients, this finding may be attributed to the limited overall sample size. Further investigation is needed to determine potential correlations between weight or hunger outcomes and HRQOL in pediatric and adolescent patients. Another limitation is the subjective nature of hunger scoring; however, there is currently no validated hyperphagia measurement for patients with BBS.

## Conclusions

BBS is a rare genetic obesity syndrome associated with clinically significant HRQOL burden for patients and their families. There is an unmet need for therapies addressing obesity and underlying hyperphagia in this population. After 1 year of treatment with setmelanotide, clinically meaningful improvements in HRQOL were observed in adult, adolescent, and pediatric patients with BBS across multiple domains, including physical function and psychosocial domains. Clinically meaningful improvement, based on IWQOL-Lite and PedsQL, was observed in 75% of patients who reported impaired HRQOL at baseline. Most patients with no impairment in HRQOL at baseline improved or preserved their health status. Clinically meaningful improvements in weight outcomes were also associated with improvements in HRQOL in adults. At the patient level, improvements were sustained over the 52-week trial period. This study highlights the need to address the high HRQOL burden experienced by patients, and further supports setmelanotide as a targeted treatment strategy for improving weight outcomes and quality of life in this population. The physical and psychosocial effects of BBS on patients and their families are complex and require comprehensive, multidisciplinary support. Additional research and experience in clinical practice is needed to support these findings.

## Data Availability

The datasets supporting the conclusions of this article are available from the corresponding author on reasonable request.

## Abbreviations

BBS: Bardet–Biedl syndrome
BMI: Body mass index
HRQOL: Health-related quality of life
IWQOL-Lite: Impact of Weight on Quality of Life Questionnaire-Lite
LEPR: Leptin receptor
MC4R: Melanocortin-4 receptor
PCSK1: Proprotein convertase subtilisin/kexin type 1
PedsQL: Pediatric Quality of Life Inventory
POMC: Proopiomelanocortin
SD: Standard deviation
